# Trends in the Laboratory Detection of Rotavirus Before and After Implementation of Routine Rotavirus Vaccination — United States, 2000–2018

**DOI:** 10.15585/mmwr.mm6824a2

**Published:** 2019-06-21

**Authors:** Benjamin D. Hallowell, Umesh D. Parashar, Aaron Curns, Nicholas P. DeGroote, Jacqueline E. Tate

**Affiliations:** ^1^Division of Viral Diseases, National Center for Immunization and Respiratory Diseases; CDC; ^2^Epidemic Intelligence Service, CDC.

Before the introduction of rotavirus vaccine in the United States in 2006, rotavirus infection was the leading cause of severe gastroenteritis among U.S. children (*1*). To evaluate the long-term impact of rotavirus vaccination on disease prevalence and seasonality in the United States, CDC analyzed national laboratory testing data for rotavirus from laboratories participating in CDC’s National Respiratory and Enteric Viruses Surveillance System (NREVSS) during the prevaccine (2000–2006) and postvaccine (2007–2018) periods. Nationally, the median annual percentage of tests positive for rotavirus declined from 25.6% (range = 25.2–29.4) in the prevaccine period to 6.1% (range = 2.6–11.1) in the postvaccine period. When compared with the prevaccine period, the postvaccine period saw declines in the annual peak in rotavirus positivity from a median of 43.1% (range = 43.8–56.3) to a median of 14.0% (range = 4.8–27.3) and in the season duration from a median of 26 weeks (range = 23–27) to a median of 9 weeks (range = 0–18). In the postvaccine period, a biennial pattern emerged, with alternating years of low and high rotavirus activity. Implementation of the rotavirus vaccination program has substantially reduced prevalence of the disease and altered seasonal patterns of rotavirus in the United States; these changes have been sustained over 11 seasons after vaccine introduction. Ongoing efforts to improve coverage and on-time vaccination (*2*) can help maximize the public health impact of rotavirus vaccination.

NREVSS is a voluntary laboratory-based passive surveillance system that collects data on eight respiratory viruses and three enteric viruses, including rotavirus (*3*). Each week, participating laboratories report to CDC the aggregate number of rotavirus tests performed and the number of those that had positive results. A reporting year begins in July (epidemiologic week 27) and ends in June (epidemiologic week 26) of the following year. Peak rotavirus activity is defined as the highest proportion of tests positive for rotavirus during a single week in a given reporting year. The beginning and end of the rotavirus season are defined as the first and last, respectively, of 2 consecutive weeks in which ≥10% of the tests are positive for rotavirus. Historically, rotavirus disease exhibited a winter-spring seasonality, with the season beginning in December–January and ending in April–May (*4*).

Results of all enzyme immunoassay (EIA) tests for rotavirus conducted during July 2000–June 2018 were obtained from laboratories participating in NREVSS. Data from the first reporting year after vaccine introduction (July 2006–June 2007), which is considered a transitional year with low vaccination coverage, were excluded from the analysis. To examine trends in rotavirus testing and detection during the prevaccine and postvaccine periods, analyses were restricted to the 23 laboratories that continuously reported rotavirus testing results for ≥26 weeks of each reporting year during July 2000–July 2018. Data were aggregated by week and are presented using a 3-week moving average for the total number of rotavirus tests performed and the number of positive test results. Trends in testing practices over time were evaluated using the Spearman rank order correlation for the annual number of tests conducted and the Cochran-Armitage test for trend for the annual proportions of tests that were positive for rotavirus. SAS software (version 9.4; SAS Institute) was used for all statistical analyses. To compare the rotavirus season, duration, and peak activity between the prevaccine and postvaccine periods, data from all reporting laboratories (annual range = 57–223) were analyzed. When analyzing the biennial trend in rotavirus seasonality, data from the first 2 reporting years after vaccine introduction (July 2006–June 2008) were excluded from the analysis.

Data from the 23 laboratories that continuously reported rotavirus testing results during 2000–2018 demonstrated a decline in both rotavirus testing and percent positivity in the postvaccine era compared with the prevaccine era (Figure 1) (Table). The number of rotavirus tests declined by approximately one third, from an annual median of 10,845 (range = 9,105–13,257) in the prevaccine era to an annual median of 7,357 (range = 4,270–11,143; p<0.001) in the postvaccine era; the number of tests positive for rotavirus declined approximately 85%, from an annual median of 2,778 (range = 2,385–3,479) in the prevaccine era to an annual median of 411 (range = 159–1,231) in the postvaccine era (p<0.001). Mirroring the trends in the number of positive tests, the median annual proportion of tests positive for rotavirus declined 76%, from 25.6% (range = 25.2%–29.4%) in the prevaccine era to 6.1% (range = 2.6%–11.1%; all p-values <0.001) in the postvaccine era.

**FIGURE 1:**
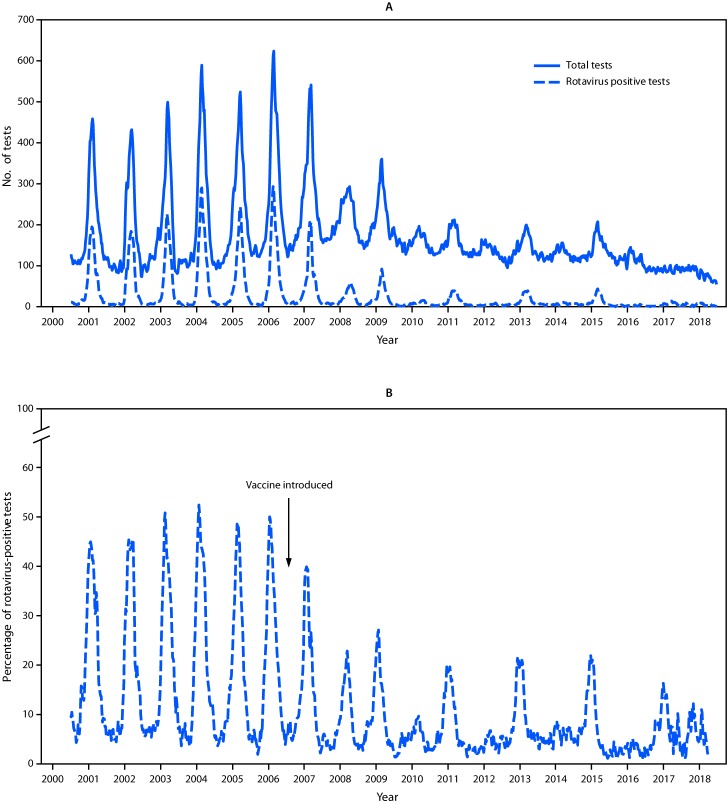
Total number of rotavirus tests and positive rotavirus tests (A) and percent positivity (B) among the 23 continuously reporting National Respiratory and Enteric Virus Surveillance System (NREVSS) laboratories* — NREVSS, United States, 2000–2018 * Data were aggregated by week and are presented using a 3-week moving average for the total number of rotavirus tests performed and the number of positive test results.

**TABLE:** Rotavirus seasonality and testing results, by years — National Respiratory and Enteric Virus Surveillance System, United States, 2000–2018.

Years	Season beginning, wk no.	Peak		Season end, wk no.	Season duration (wks)	No. of rotavirus tests performed*	Positive rotavirus tests* No. (%)	% Change in rotavirus tests performed*^,§^	% Decline in positive rotavirus tests*
		Wk no.	% Positive tests						
2000–2006^†^	50	9	43.1	24	26	10,845	2,778 (25.6)	Referent	Referent
2007–2008	9	17	17.3	21	12	11,143	1,034 (9.3)	2.7	62.8
2008–2009	4	11	25.3	21	17	11,078	1,231 (11.1)	2.1	55.7
2009–2010	—^¶^	18	10.9	—	—	8,345	411 (4.9)	−23.1	85.2
2010–2011	3	11	23.4	21	18	8,152	734 (9.0)	−24.8	73.6
2011–2012	—	22	12.2	—	—	7,129	244 (3.4)	−34.3	91.2
2012–2013	1	13	27.3	18	17	7,357	718 (9.8)	−32.2	74.2
2013–2014	—	21	11.3	—	—	6,687	352 (5.3)	−38.3	87.3
2014–2015	3	11	25.1	16	13	7,448	724 (9.7)	−31.3	73.9
2015–2016	—	20	4.8	—	—	6,145	159 (2.6)	−43.3	94.3
2016–2017	9	13	21.7	19	10	4,708	287 (6.1)	−56.6	89.7
2017–2018	—	17	10.3	—	—	4,270	235 (5.5)	−60.6	91.5

* Testing data from the 23 laboratories that continuously reported rotavirus test results during 2000–2018.

^†^ Median data are reported for the prevaccine seasons spanning 2000–2006.

^§^ Compared with number of tests performed during 2000–2006.

^¶^ Dashes indicate not applicable because seasonal start and end thresholds were not reached.

Analysis of data from all reporting laboratories indicated that rotavirus test positivity during peak activity declined by approximately two thirds, from an annual median of 43.1% (range = 43.8–56.3) in the prevaccine era to 14.0% (range = 4.8–27.3) in the postvaccine era (Table). In addition, in the postvaccine era, the rotavirus season began later in the year, and the annual median season duration was reduced from 26 weeks (range = 23–27) in the prevaccine era to 9 weeks (range = 0–18) in the postvaccine era.

In the postvaccine period, a biennial pattern emerged, with alternating years of low and high rotavirus activity (Figure 1). In low-activity years after vaccine introduction, the 10% test positivity threshold for the start of the season was never reached, peak activity occurred later in the year (weeks 17–22), and the median peak activity was 8.7% (range = 4.8–12.2) (Figure 2). In contrast, in the high-activity years after vaccine introduction, the median season duration was 17 weeks (range = 10–18), peak activity occurred earlier in the year (weeks 11–13), and the median peak test positivity was 25.1% (range = 21.7%–27.3%). Over time, rotavirus activity and seasonality have remained relatively consistent in the low- and high-activity years; however, the season duration during high-activity years has slowly decreased.

**FIGURE 2:**
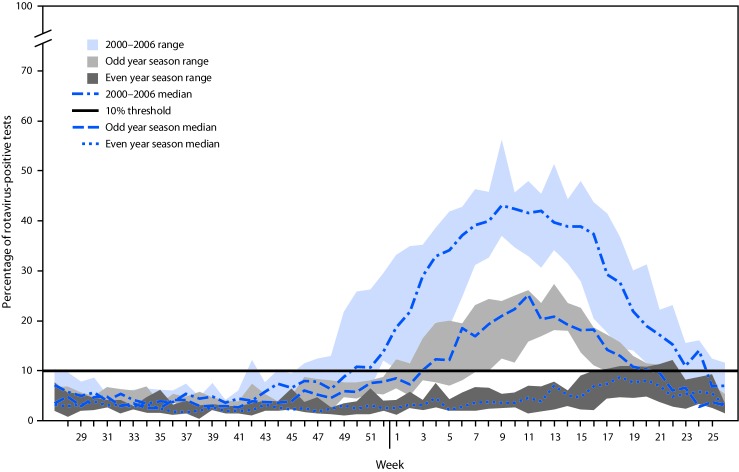
Rotavirus season duration and peak activity for prevaccine (2000–2006) and postvaccine years (2008–2018), stratified by even/odd year season,* by week of season — National Respiratory and Enteric Virus Surveillance System, United States 2000–2018 * Odd year seasons: 2008–09; 2010–11; 2012–13; 2014–15; 2016–17. Even year seasons: 2009–10; 2011–12; 2013–14; 2015–16; 2017–18.

The number of laboratories reporting EIA test results for rotavirus to NREVSS in the postvaccine era has gradually declined over time, from a high of 223 laboratories reporting 29,198 EIA test results (2008–09 season) to 109 laboratories reporting 14,737 EIA test results (2017–18 season). However, in recent years, the number of laboratories reporting polymerase chain reaction (PCR) tests for rotavirus has increased, from 19 laboratories reporting 16,490 PCR test results (2014–15 season) to 80 laboratories reporting 87,775 PCR test results (2017–18 season).

## Discussion

Implementation of the rotavirus vaccination program has markedly reduced the prevalence of rotavirus disease in the United States. In all postvaccine-era seasons from 2007–08 to 2017–18, rotavirus activity consistently fell below the 2000–2006 baseline, and seasons were shorter in duration compared with those during the prevaccine era, which further attests to the long-term benefits of the rotavirus vaccination program. Some of the observed changes in rotavirus activity that occurred after vaccine introduction could be due to concurrent changes in rotavirus testing practices as PCR-based multipathogen detection assays are increasingly used. Although this shift in testing practices might explain some of the declines observed in the total number of tests performed and the number of positive tests, the substantial reductions in the proportion of rotavirus tests that were positive (which is less affected by changes in testing practices alone), supports attribution of the declines to the effects of vaccination.

Introduction of rotavirus vaccine has also modified the seasonality of rotavirus disease in the United States, with a biennial trend emerging in the postvaccine era beginning in the 2008–09 rotavirus season. Since vaccine introduction, coverage has slowly increased and completed coverage has plateaued at approximately 70%. The lower coverage of rotavirus compared with other childhood vaccines might be explained in part by the fact that rotavirus vaccine does not offer the same opportunity for catch-up because the first dose must be given by age 15 weeks, and the series must be completed by age 8 months (*2*,*5*). The biennial trend observed in the United States could be attributed to this low vaccination coverage, with the number of susceptible children accumulating in low rotavirus activity years, resulting in a higher number of susceptible children and a subsequent rotavirus outbreak during the following season (*6*). Countries that rapidly achieved and maintained rotavirus vaccination coverage of 90%–95%, such as the United Kingdom, have experienced a sustained decline in rotavirus activity without the biennial trend observed in the United States (*7*). As vaccination coverage and on-time vaccination continue to improve in the United States, the seasonality of rotavirus disease can be monitored to see whether the biennial trend continues.

The findings in this report are subject to at least three limitations. First, aggregate NREVSS data are reported, without demographic or clinical characteristics (including vaccination status), precluding examination of these characteristics. Second, these data were collected from a passive surveillance system composed of a convenience sample of laboratories and might not be representative of all those in the United States. Finally, because rotavirus testing does not affect clinical management (which focuses on rehydration and syndromic management), testing practices vary from site to site and year to year, which might affect data comparability (*1*,*6*). However, NREVSS data have advantages, including the ability to describe trends in rotavirus activity in the United States in a timely fashion. In addition, NREVSS data have consistently aligned with rotavirus-related U.S. hospital discharge data and active surveillance data (*8*–*10*).

Rotavirus vaccination has resulted in a significant and sustained reduction of disease prevalence and has modified the seasonality of rotavirus disease in the United States. To maximize the public health impact of rotavirus vaccination, efforts to improve coverage and on-time vaccination should continue.

**Summary**

**What is already known about this topic?**

Before the introduction of rotavirus vaccine in the United States in 2006, rotavirus infection was the leading cause of severe gastroenteritis among U.S. children.

**What is added by this report?**

Implementation of the U.S. rotavirus vaccination program reduced the annual proportion of positive rotavirus tests, reduced peak rotavirus activity, and shortened the duration of the rotavirus season. Biennial seasonal patterns that emerged after vaccine introduction have continued with alternating years of low and high rotavirus activity.

**What are the implications for public health practice?**

Ongoing efforts to improve coverage and on-time vaccination can help maximize the public health impact of rotavirus vaccination.
